# Vitamin B_12_ in Health and Disease

**DOI:** 10.3390/nu2030299

**Published:** 2010-03-05

**Authors:** Fiona O’Leary, Samir Samman

**Affiliations:** Discipline of Nutrition and Metabolism, School of Molecular Bioscience, University of Sydney, NSW 2006, Australia; Email: fiona.oleary@usyd.edu.au

**Keywords:** vitamin B_12_, physiology, nutrition, adults, chronic disease

## Abstract

Vitamin B_12_ is essential for DNA synthesis and for cellular energy production.This review aims to outline the metabolism of vitamin B_12_, and to evaluate the causes and consequences of sub-clinical vitamin B_12_ deficiency. Vitamin B_12_ deficiency is common, mainly due to limited dietary intake of animal foods or malabsorption of the vitamin. Vegetarians are at risk of vitamin B_12_ deficiency as are other groups with low intakes of animal foods or those with restrictive dietary patterns. Malabsorption of vitamin B_12_ is most commonly seen in the elderly, secondary to gastric achlorhydria. The symptoms of sub-clinical deficiency are subtle and often not recognized. The long-term consequences of sub-clinical deficiency are not fully known but may include adverse effects on pregnancy outcomes, vascular, cognitive, bone and eye health.

Vitamin B_12_ deficiency was first described in 1849, and was considered to have a fatal outcome until 1926 when a diet of liver, high in vitamin B_12_, was shown to slow the disease process. Much is now known about the biochemistry and metabolism of vitamin B_12_, however, the diagnosis of its deficiency has become more complicated with the classification of a “sub-clinical” deficiency category, characterized by serum vitamin B_12_ concentrations that were once considered to be adequate. Vitamin B_12_ deficiency was previously thought to take many years to develop, and only in strict vegetarians or those with pernicious anaemia. More recent research has suggested that there are disease implications associated with sub-clinical B_12_ deficiency, which develop most commonly due to malabsorption or dietary inadequacy. The rates of sub-clinical deficiency of vitamin B_12_ are high in developing countries, in the elderly, and in vegetarian populations. The long term consequences are not fully known but may include adverse effects on pregnancy outcomes and aspects of ageing.

## 1. Vitamin B_12_ Function

Vitamin B_12_ also known as cobalamin, comprises a number of forms including cyano-, methyl-, deoxyadenosyl- and hydroxy-cobalamin. The cyano form, which is used in supplements, is found in trace amounts in food [1]. The other forms of cobalamin can be converted to the methyl- or 5-deoxyadenosyl forms that are required as co factors for methionine synthase and L-methyl-malonyl-CoA mutase.

Methionine synthase is essential for the synthesis of purines and pyrimidines. The reaction depends on methyl cobalamin as a co-factor and is also dependent on folate, in which the methyl group of methyltetrahydrofolate is transferred to homocysteine to form methionine and tetrahydrofolate. A deficiency of vitamin B_12_ and the interruption of this reaction leads to the development of megaloblastic anaemia. Folate deficiency independent of vitamin B_12_ also causes megaloblastic anaemia [2]. Methylmalonyl CoA mutase converts methylmalonyl CoA to succinyl CoA, with 5-deoxy adenosyl cobalamin required as a cofactor. It is a defect in this reaction, and the subsequent accumulation of methylmalonyl CoA that is thought to be responsible for the neurological effects in vitamin B_12_ deficiency [2].

Serum vitamin B_12_ is bound to proteins known as transcobalamins (TC). The majority of the vitamin, approximately 80%, is transported on the inactive TCI (also called haptocorrin). The active transport protein for vitamin B_12_ is transcobalamin II (TCII), which caries about 20% of the vitamin in the circulation [3]. Holo-transcobalamin (holo-TC) is TCII with attached cobalamin, which delivers vitamin B_12_ to cells. A low serum vitamin B_12_ concentration can be associated with a deficiency of TCI, while TCII levels and so vitamin B_12_ status remain adequate [4].

## 2. Biochemical Assessment of Vitamin B_12_ Status

Traditionally vitamin B_12_ status is assessed by its concentrations in serum, however, concerns have been raised about the use of serum vitamin B_12_ measurements alone. Although low serum vitamin B_12_ concentrations are a sensitive indicator of vitamin B_12_ deficiency and high vitamin B_12_ concentrations generally indicate sufficiency, the interpretation of the intermediate range of vitamin B_12_ concentrations is unclear [4].

Methylmalonic acid (MMA) and homocysteine (tHcy) are recognized indicators of vitamin B_12_ status. Their measurement has highlighted the existence of sub-clinical deficiency, the consequences of which are still being elucidated. MMA is considered to be the specific indicator of cobalamin metabolism, and tHcy is raised in vitamin B_12 _deficiency along with deficiencies of folate and vitamin B_6_. These biomarkers can be confounded by physiological or environmental conditions. Plasma tHcy concentrations are elevated also with renal impairment, polymorphisms in methylenetetrahydrofolate reductase (MTHFR), or the use of some medication. Plasma MMA concentrations are elevated also in renal insufficiency, common in older people [4,5]. 

It has been recommended by some authors [6,7] that measuring serum vitamin B_12_ concentrations and following up low values with MMA measurements is an appropriate strategy for the assessment of vitamin B_12_ status. However, the threshold of vitamin B_12_ at which further testing should occur is controversial. A study of serum vitamin B_12_, MMA and tHcy concentrations indicates that if a lower limit of normal (200 ng/L or 147 pmol/L) is used, patients with increased MMA would be missed, however, if higher values (500 ng/L or 370 pmol/L) are used, most patients would need follow-up MMA tests which may be within the normal range [8]. Carmel recommends a composite criteria based on serum vitamin B_12_ < 148 pmol/L, or 148–258 pmol/L and MMA > 0.30μmol/L, or tHcy > 13 nmol/L (females) and >15 nmol/L (males) be used to define inadequate vitamin B_12_ status [9].

Studies that have assessed the use of holo-TC as a marker of vitamin B_12_ status show similarity in specificity and sensitivity to serum vitamin B_12_ concentrations. However, when used in combination with vitamin B_12_ the predictive value for determining vitamin B_12_ deficiency is improved [10].

## 3. Absorption

Vitamin B_12_ is bound to protein in food and is available for absorption after it has been cleaved from protein by the hydrochloric acid produced by the gastric mucosa. The released cobalamin then attaches to R protein and passes into the duodenum where the R protein is removed and free cobalamin binds to Intrinsic Factor (IF). The IF-cobalamin complex is absorbed by the distal ileum and requires calcium [2]. Vitamin B_12_ enters the circulation about 3–4 hours later bound to TC. 

Vitamin B_12_ is secreted in bile and reabsorbed via the enterohepatic circulation by ileal receptors which require IF, thus the development of vitamin B_12_ deficiency is likely to be more rapid in patients with pernicious anaemia as IF is lacking [3]. Vitamin B_12_ is excreted via the faeces, which is composed of unabsorbed biliary vitamin B_12_, gastrointestinal cells and secretions, and vitamin B_12_ synthesised by bacteria in the colon. It is estimated that daily vitamin B_12_ losses are in proportion to body stores with approximately 0.1% excreted per day [11]. Excessive vitamin B_12_ in the circulation, e.g., such as after injections, usually exceeds the binding capacity of TC and is excreted in the urine [3]. 

Historically, vitamin B_12_ absorption has been measured by a number of methods including whole body counting of radiolabeled vitamin B_12_, metabolic balance studies [1] or controlled feeding studies in vitamin B_12_-depleted individuals [12]. 

It is known that the total amount of vitamin B_12_ that is absorbed increases with vitamin B_12_ intake but that the percentage absorption decreases with increasing doses [13]. One study using crystalline vitamin B_12_ supplements reported that 50% was retained at a 1 µg dose, 20% at a 5 µg dose and 5% at a 25 µg dose, suggesting saturation of the absorption mechanisms [14]. The absorption capacity is thought to recover to baseline levels within 4-6 hours allowing for efficient absorption of the next dose [11]. Approximately 1% of large doses of crystalline vitamin B_12_ found in some supplements (1,000µg), are absorbed through a mass action process, even in the absence of IF [15], indicating crystalline vitamin B_12_ in high doses and food vitamin B_12_ are absorbed by different mechanisms.

The Schilling test was the classical procedure for assessing the absorption of vitamin B_12_ but is now rarely used. As there has been no replacement a number of individual tests must be used to diagnose the cause of vitamin B_12_ deficiency. Tests that diagnose atrophic gastritis, a common cause of vitamin B_12_ malabsorption, include gastroscopy or serum gastrin and pepsinogen levels. Specific tests for pernicious anaemia include IF antibodies and serum gastrin estimation. MMA and tHcy are better markers of vitamin B_12_ status, although they are not appropriate for testing absorption [16]. An overview of the medical management of vitamin B_12_ deficiency can be found in a recent article by Ralph Carmel [17].

## 4. Food Sources and Bioavailability of Vitamin B_12_

Vitamin B_12_ is synthesised by certain bacteria in the gastrointestinal tract of animals and is then absorbed by the host animal. Vitamin B_12_ is concentrated in animal tissues, hence, vitamin B_12_ is found only in foods of animal origin [11]. Foods that are high in vitamin B_12_ (µg/100g) include: liver (26–58), beef and lamb (1–3), chicken (trace-1), eggs (1–2.5) and dairy foods (0.3–2.4).

There are no naturally occurring bioactive forms of vitamin B_12_ from plant sources. Some plant foods contain added vitamin B_12_ and others e.g., seaweed and mushrooms contain vitamin B_12_ analogues that are inactive in humans, although 2 studies suggest certain types of Japanese seaweed (nori) have prevented vitamin B_12_ deficiency in vegans [18]. Some foods that are contaminated or fermented by bacteria e.g., tempeh and Thai fish sauce, have been reported to contain vitamin B_12_[18], although these may have low affinity with IF and may be poorly absorbed [19].

A number of methods have been used to determine the vitamin B_12_ content of foods. Microbiological assays using vitamin B_12_ requiring bacteria were used, however, they are no longer the reference method as measurement uncertainty is high. Radio isotope dilution assays with labeled vitamin B_12_ and hog IF are used [20]. Further advances are expected with the development of more specific monoclonal antibodies tests using specific binding proteins [21].

The bioavailability of vitamin B_12_ in humans is dependent on an individual’s gastrointestinal absorption capacity. As outlined previously, vitamin B_12_ absorption is complex and there are adverse changes with age. In view of the technical challenges and biological factors, there is little data on the bioavailability of dietary vitamin B_12_ in humans. It is thought that 1.5–2.0 µg of synthetic vitamin B_12_ saturates the IF-cobalamin ileal receptors, but other studies have shown higher absorption rates [1,11]. In normal humans the absorption of vitamin B_12_ from foods has been shown to vary depending on the quantity and type of protein consumed [19]. Vitamin B_12_ from foods appear to have different absorption rates with better absorption from chicken and beef as compared to eggs. Studies assessing absorption of food bound vitamin B_12_ from whole foods are described in Table 1. 

## 5. Vitamin B_12_ Requirement

The Recommended Dietary Intake (RDI) is set to prevent megaloblastic anaemia and maintain adequate serum vitamin B_12_ concentrations. It is assumed that 50% of dietary vitamin B_12_ is absorbed. The RDI and estimated average requirement (EAR) do not vary once adulthood is reached. However, the US and Australian Nutrient Reference Values suggest that older adults with atrophic gastritis may require higher intakes of vitamin B_12_-rich foods, vitamin B_12_ fortified foods or supplements [3,22]. The US Institute of Medicine has recommended that adults over 51 years consume most of their vitamin B_12_ from fortified foods or from supplements, again recognising the high rates of malabsorption due to gastritis that occurs with age. Vitamin B_12_ stores last several years and the development of deficiency is slow, however the combination of malabsorption and inadequate dietary intake will hasten deficiency [3]. 

**Table 1:** Bioavailability of vitamin B_12_ from whole foods.

Food Type	Subjects	Vitamin B_12 _content	% Absorption, mean (range)	Analysis method	Reference
Mutton	3 healthy young subjects	0.9 µg in 100g portion	65 (56–77)	Radiolabelled vitamin B_12_ , whole body counting	[11]
	2 healthy young subjects	3.03 µg in 200g portion	83		
	2 healthy young subjects	5.11 µg in 300g portion	53		
Liver pate	6 healthy subjects	38 µg per serve	9.1 (5.1–19.5)	Radiolabelled vitamin B_12_ , whole body counting	[11]
	4 older subjects		4.5 (2.4–6.0)		
	5 subjects with pernicious anaemia		1.8 (0–3.7)		
Chicken	3 healthy subjects	0.4–0.6 µg in 100g portion	65 (58–74)	Radiolabelled vitamin B_12_ , faecal excretion studies	[23]
		0.8–1.3 µg in 200g portion	63 (48–76)		
		1.3–1.9 µg in 300g portion	61 (49–75)		
Fish	3 healthy subjects	2.1 µg in 50g portion	42	Radiolabelled vitamin B_12_ , faecal and urinary excretion studies	[24]
		3.1 µg in 100g portion	38		
		9.2 µg in 200g portion	42		
		13.3 µg in 300g portion	30		
Eggs: boiled, scrambled, fried	18 healthy subjects	0.9–1.4 µg in 100g portion	3.7–9.2	Radiolabelled vitamin B_12_ , faecal and urinary excretion	[25]

## 6. Vitamin B_12_ Deficiency

Deficiency is usually caused by the malabsorption of vitamin B_12_ although dietary inadequacy is common in the elderly, vegans or ovo-lacto vegetarians with poor diets. Causes can also relate to inadequate IF production, atrophic gastritis, interference with the ileal uptake of vitamin B_12_ due to disease, resection or interference by bacterial overgrowth, drug-nutrient interactions as well as some less common genetic defects [3]. 

Vegans who consume no foods of animal origin can meet their vitamin B_12_ requirement from fortified foods or supplements. Ovo-lacto vegetarians with only a small intake of dairy foods or eggs, may require supplemental vitamin B_12_. Pregnant and/or lactating women following vegetarian or vegan diets are at high risk of deficiency due to the increased metabolic demand for vitamin B_12_ and require adequate intake of vitamin B_12_-containing foods or supplements. 

The elderly are at risk of undernutrition in general, predominately due to reduced intake related to illness but also due to physical capacity e.g., difficulties with food preparation, and psychological factors e.g., depression. Protein bound malabsorption is thought to be the most common cause of sub-clinical vitamin B_12_ deficiency in the elderly and is commonly associated with some degree of atrophic gastritis. Gastritis or inflammation of the gastric mucosa increases with age and results in a reduction, or in some cases, complete loss of the acid required to cleave vitamin B_12_ from protein. Synthetic vitamin B_12_ remains available for absorption as it is not protein bound [3,22].

Pernicious anemia is the end stage of an auto-immune gastritis and results in the loss of synthesis of IF. It is this loss of IF that causes vitamin B_12_ deficiency and if untreated, megaloblastic anaemia and neurological complications develop. Pernicious anaemia is treated with vitamin B_12_ injections, or large doses of oral vitamin B_12_. Vitamin B_12_ deficiency will also develop after gastric antrum resection as this is the site of secretion of IF and acid [3]. 

Reduced ileal uptake of vitamin B_12_ can be caused by competition for vitamin B_12_ in patients with bacterial overgrowth or parasitic infection. Resection or diseases of the ileum such as Crohn’s Disease or other chronic bowel inflammatory conditions also cause malabsorption of vitamin B_12_[3].

The potential masking of vitamin B_12_ deficiency by folate fortification of the food supply has also raised some safety concerns. When vitamin B_12_ concentrations are low, high doses of folate (supplements or food fortification) allow DNA synthesis to continue, prevent megaloblastic anaemia and potentially “mask” vitamin B_12_ deficiency, potentially allowing homocysteine and MMA concentrations to rise and neurological damage to progress. Neurological damage in the absence of anaemia has been reported in 20-30% of cases of vitamin B_12 _deficiency [4]. In view of this and the effect of vitamin B_12_ deficiency on pregnancy outcomes [26,27], there is discussion of the need to fortify flour with vitamin B_12_. Vitamin B_12_ fortification of flour is most likely to benefit those with poor dietary intake of vitamin B_12_ and the elderly with food bound malabsorption, but would be inadequate for those with pernicious anaemia, which affects 2–4% of the US population depending on ethnicity [28]. Patients with pernicious anaemia require larger oral supplements (e.g., 500–1,000 µg/d) or intramuscular injections. In developing countries, fortification could potentially have a more significant impact as the population’s intake is often low. However, as yet there not enough intervention trials on the effect of different fortification levels of flour in different populations [29].

## 7. Drug-nutrient Interactions

Some medications are thought to interfere with the absorption or metabolism of vitamin B_12_. These include proton pump inhibitor (PPI) medications, metformin, nitrous oxide anaesthesia, some epileptic medications and colchicine.

PPI medications are commonly used in the elderly for the treatment of gastro-oesophageal reflux disease. PPI medications act by reducing the secretion of gastric acid and pepsin, theoretically leading to a decrease in the absorption of protein-bound vitamin B_12_. However, the current literature on PPI usage and vitamin B_12_ status is inconsistent [30,31,32,33]. The monitoring of vitamin B_12_ concentrations is recommended for patients undergoing prolonged PPI treatment, in recognition that the bioavailability of food-bound vitamin B_12_ may be compromised [3]. 

Metformin is a biguanide used for the treatment of non-insulin dependent diabetes and some patients taking this medication develop megaloblastic anaemia [34,35]. This may relate to intestinal mobility changes or bacterial overgrowth competing for vitamin B_12_ in the gastrointestinal tract. It has also been shown that calcium improves the uptake of vitamin B_12_ in metformin users [35].

Nitrous oxide anesthesia inhibits methionine synthase and L-methylmalonyl–CoA mutase and produces deficiency symptoms despite concentrations of serum vitamin B_12_ in the normal range [36]. Antiepileptic drugs have been associated with low concentrations of vitamin B_12_, but this is controversial with some studies showing no change and others increased levels of vitamin B_12_[37].

## 8. Vitamin B_12_ and Neural Tube Defects (NTD)

NTD include spina bifida, anencephaly, and encephalocele. These are caused by the failure of the neural tube to close during gestation. The aetiology of NTD is not fully understood but risk factors include folate deficiency, genetic and environmental factors [26,27]. A significant reduction in NTD has been reported since folate fortification of the US food supply [38]. As folate has reduced but not eliminated rates of NTD, research is ongoing to determine further strategies to minimise risk. Low vitamin B_12_ status has been postulated as a potential risk factor for NTD [39] since vitamin B_12_ acts as a cofactor for methionine synthase in the folate cycle. When vitamin B_12_ supply is low, the folate needed for DNA synthesis remains trapped in the methylation cycle and cell replication is impaired. Studies assessing the impact of vitamin B_12_ status on NTD are shown in Table 2. The studies consistently report a 2-4 fold increased risk of NTD with low vitamin B_12_ status. The studies were undertaken in a range of population groups including those that are exposed to folate fortified foods, as well and non-fortified populations.

**Table 2:** Case control and cohort studies of vitamin B_12_ and Neural Tube Defects.

Design and reference	Study Details	Main Outcome
Case control [39]	81 NTD cases and 247 controls	In cases only, plasma vitamin B_12_ and plasma folate affected maternal Red Cell Folate (multiple r = 0.68, p < 0.001).
Case control [40]	84 NTD pregnancies and 110 controls	Women with lower vitamin B_12_ have increased risk of NTD.
Cohort [41]	Vitamin B_12_ at 15 weeks' gestation	Vitamin B_12_ <185 pmol/L associated with the highest risk of NTD.
Case control [42]	46 NTD pregnancies and 44 controls	Lower serum vitamin B_12_ (p = 0.005) in cases compared to controls
Case control [43]	35 NTD neonates and parents vs 24 normal neonates.	Low vitamin B_12_ in both the parents of child with NTD.
Case control [44]	89 NTD and 422 controls	Increased NTD risk with lower holo-TC.
Case control ^1^[45]	36 NTD vs normal pregnancy.	Low vitamin B_12_ associated with 2-3 x increased risk for NTD
Case control [46]	46 NTD and 73 control mothers	For NTD holo-TC % (holo-TC/total TCII ) Q1vs Q4 OR = 5.0 (95% CI:1.3, 19.3).
Case control ^1^[47]	57 NTD cases and 186 controls	Q1 vs Q5 of vitamin B_12_ OR = 3.0 (95% CI:1.4, 6.3)
Case control ^1^[48]	45 mothers and NTD children vs 83 controls	Case mothers with vitamin B_12_ ≤ 185 pmol/L OR = 3.5-fold (95% CI:1.3, 8.9) for NTD risk.
Case control [49]	56 NTD babies and mothers vs 97 control children and mothers.	Low vitamin B_12 _levels increase risk of NTD.
Case control ^1^[50]	60 NTD cases and 94 controls	NTD for mothers for vitamin B_12_ levels ≤ 5^th ^% *vs* ≥95^th^
Case control [51]	32 NTD pregnancies and 132 control pregnancies.	MMA higher in cases vs controls.

^1^Study performed in folate fortified population, NTD = Neural tube defects, OR (95%CI) = Odds Ratio and 95% confidence interval, Q4 = 4^th^ quartile, Q5 = 5^th^ quintile, RBC = red blood cell, tHcy = total homocysteine concentration, holo-TC = holotranscobalamin II, total TCII = total transcobalamin II, MMA = methylmalonic acid

## 9. Vitamin B_12_ and Cardiovascular Disease (CVD)

Nutritional risk factors for CVD include hypercholesterolaemia, hypertension and obesity. Elevated tHcy concentrations are also considered a risk factor, however, it is unclear if tHcy is a modifiable risk factor or an independent marker of the disease process. Much of the research into CVD and tHcy is related to the effects of folate supplementation with or without the addition of vitamins B_12_ and B_6_. Investigations of the relationship between CVD and vitamin B_12_ per se are limited.

Meta-analyses of prospective studies (Table 3) have consistently shown associations between tHcy and increased risk of CVD. Supplementation with vitamin B_12_ of doses ranging from 0.02–1 mg/d produces approximately 7% reduction in tHcy, while folate produces 10–30% reduction in risk. Vitamin B_6_ has been shown to have little effect [52].

**Table 3:** Meta-analyses of studies assessing vitamin B_12_ and CVD.

Trial Type	Study Details	Main Outcome
Meta-analysis [53]	9 case-control studies. Assessed associations between tHcy and CVD risk.	5µM tHcy increment associated with increased risk of CAD, OR = 1.6 (95% CI:1.4 to 1.7) for males and 1.8 (95% CI:1.3 to 1.9) for females.
Meta-analysis [54]	30 prospective or retrospective studies assessed tHcy and CVD risk.	25% lower tHcy associated with lower risk of IHD & stroke.
Meta-analysis 7 RCTs [55]	B vitamin supplementation and tHcy lowering, assessed effect of vitamin B_12 _(range 0.02–1.0 mg/day)	Vitamin B_12_ (median dose 0.4 mg/d) - further decrease (-7%) in tHcy
Meta-analysis 12 RCTs [56]	Preexisting CVD or renal disease- included 3 studies of vitamin B_12 _supplementation, with doses 0.4–1.0 mg B_12_/day.	Reduction in stroke risk in vitamin B_12_ (1 mg/d) intervention OR = 0.76 (95% CI:0.59, 0.96)
Meta-analysis8 RCTs [57]	4 studies assessed vitamin B_12_ supplementation (0.018–1 mg) and stroke risk	Reduction in stroke greater in longer trials with more tHcy lowering and no stroke history. No specific effect of vitamin B_12_.
Meta-analysis 24 RCTs [58]	Assessed CIMT: 3 with vitamin B_12_: 0.4–0.5 mg/d; endothelial function: 5 with B_12_: 6 µg–1 mg/day	↓ CIMT, ↑ FMD found in short-term not long term trials

µM = micromolar, tHcy = total homocysteine, CAD = coronary artery disease, OR = odds ratio, CI=confidence intervals, CVD = coronary vascular disease, IHD=ischaemic heart disese, CIMT = carotid intima media thickness, FMD = flow mediated dilation

The recent B vitamin supplementation trials investigating the effect of tHcy reduction and CVD did not show the expected reductions in risk of CVD [59,60,61,62,63]. All of these randomised controlled trials (RCTs) included vitamin B_12_ supplementation (ranging from 6 µg-1 mg) in tandem with folate, and it is not possible to determine the individual impact of vitamin B_12_. A number of reviews have discussed the limitations of these trials [64,65,66] and identified inadequate treatment with vitamin B_12_ as one of the limitations.

Subgroup analysis of the VISP Trial found that patients with higher baseline vitamin B_12_ concentrations, taking high dose vitamins, had the best outcomes and those with lower baseline vitamin B_12_ taking low-dose vitamins had the poorest outcomes for stroke, death, and coronary events, suggesting higher vitamin B_12_ doses may be needed in some patients [67]. Vitamin B_12_ has been shown to be a major determinant of tHcy concentrations in subjects with adequate folate status [68] and the existence of vitamin B_12_ deficiency could be one reason for the lack of effect of intervention with folate [69].

## 10. Cognitive Decline

The assessment of vitamin B_12_ status forms part of the screening process for dementia, however, the effects of sub-clinical levels of vitamin B_12_ on cognitive status are unclear. Studies investigating cognitive decline and vitamin B_12_ status using serum vitamin B_12_ concentrations alone, have been inconclusive [70]. Raised MMA concentrations are associated with cognitive decline and Alzheimer’s Disease [71]. It has been suggested that holo-TC and MMA and the ratio holo-TC:vitamin B_12 _[72,73] are better correlated with cognition and the rate of cognitive decline in elderly subjects. In older people with low vitamin B_12_ status, a high serum folate concentration was associated with increased odds of cognitive impairment, but in subjects with normal vitamin B_12_ status, high serum folate was found to be protective against cognitive impairment [74].

To date, there are few intervention studies that examine the relationship between vitamin B_12_ and cognitive function. A Cochrane review, based on 2 studies, identified no effect of supplementation with vitamin B_12_ alone on cognitive score in older adults [75]. A meta-analysis and review identified a correlation between tHcy and Alzheimer’s Disease, and suggested the effect was due to lower levels of vitamins B_12_, B_6 _and folate [76]. These studies suggest a role for vitamin B_12_ in the prevention of cognitive decline. However, more long-term studies using biomarkers of vitamin B_12_ status and intervention studies from mid-life are needed to determine the effects of B vitamins on cognition.

## 11. Osteoporosis

Dietary factors associated with the development of osteoporosis include inadequate protein, calcium and vitamin D. More recently, there has been interest in the effect of other nutrients, including vitamin B_12_ on bone health.

Elevated tHcy has been associated with an increased risk of bone fractures, however it is not clear whether this is related to tHcy per se, to the level of vitamins B_12_, B_6_ or folate which are required for its metabolism, or to other causes of elevated tHcy such as environmental factors or underlying disease. A recent systematic review found that there is evidence for the association between tHcy and increased fracture risk, but less conclusive evidence for tHcy and low bone mineral density (BMD) or for the association between vitamin B_12_ and either fracture risk or low BMD [77]. Intervention trials of the association between B vitamin supplementation have also shown mixed results.

Positive effects of the supplementation of B vitamins on BMD have been found in a subgroup of osteoporotic patients with high tHcy and stroke patients at risk for osteoporosis [78,79], but none in a group of healthy older people or from the secondary analysis of the HOPE Trial for CVD reduction [80,81]. Cohort studies with more than 1,000 subjects or smaller intervention trials are summarized in Table 4. Some of the inconsistencies in study results may be due to differences in the study populations [82,83], differences in the cut points used to define vitamin B_12_ status, and the reliance on serum vitamin B_12_ concentrations rather than more specific biomarkers.

**Table 4:** Studies of vitamin B_12_ and risk of osteoporosis or fracture.

Design and reference	Study Details	Main Outcome	Reduced risk
Cohort [84]	Elderly, fracture risk	Low vitamin B_12_ and/or HHcy: RR = 3.8 (95% CI:1.2, 1.6) males and 2.8 (95% CI:1.3, 5.7) females	Yes
Cohort [85]	Elderly, fracture risk	tHcy > 14, hip fracture HR = 1.49; (95% CI: 0.91, 2.46)	No
Cohort [86]	Hip fracture risk	fracture for high vs low tHcy (≥15 *vs* <9 µM), HR=2.42 (95% CI:1.43, 4.09) in women	Yes
Cohort [87]	Elderly, fracture risk	For 1 SD in tHcy fracture RR =1.4 (95% CI:1.2, 1.6)	Yes
Cohort [88]	Elderly BMD, tHcy, MTHFR polymorphisms	OR for low BMD w HHcy ≥15 µM *vs.* low tHcy OR=1.96 (95% CI:1.40, 2.75) for females.	Yes
Cohort [89]	Elderly BMD and plasma vitamins	Vitamin B_12_ <148 pM had lower BMD at hip (males) and spine (females) p < 0.05.	Yes
Cohort [90]	Elderly subjects (n=1550)	Serum vitamin B_12_ <15^th^ percentile: OR of osteoporosis/osteopenia = 2.0 (95% CI:1.0, 3.9).	Yes
RCT [79]	559 subjects:5 mg folate, 1.5 mg vitamin B_12_ or placebo	RR for hip fracture = 0.20 (95% CI: 0.08, 0.50)	Yes
RCT [78]	47 Osteoporotic subjects 2.5 mg folate, 0.5 mg vitamin B_12_ and 25 mg B_6_ or placebo.	No changes in BMD or bone metabolism markers.	No
RCT [80]	Healthy older n = 276; folate 1 mg, vitamin B_12_ 0.5 mg, B_6 _10 mg or placebo.	No differences in bone markers in vitamin vs placebo groups.	No
CT [81]	5522 subjects with vascular disease, 2.5 mg folic acid, 50 mg B_6_, 1 mg vitamin B_12_ or placebo	HR =1.06 (95% CI:0.81, 1.40) for fracture risk in supplemented vs non supplemented	No

HHcy = hyperhomocysteinaemia, tHcy = total homocysteine, CI = confidence intervals, SD = standard deviation, RR = relative risk, OR = odds ratio, HR = hazard ratio.

## 12. Other Aspects of Vitamin B_12_ and Ageing

Vitamin B_12_ has been associated with the development of age related macular degeneration (AMD) and risk of frailty, both leading causes of disability in the elderly.

AMD is the leading cause of vision loss in the elderly. Risk factors include increasing age, family history, hypertension, smoking, obesity, sunlight exposure and hypercholesterolemia [91]. Some [91,92] but not all [93] cross sectional studies have found lower vitamin B_12_ concentrations in AMD cases. However, a recent RCT with 5205 female health professionals at risk of vascular disease found a 34% reduction in the relative risk of AMD after supplementation with vitamins B_12_, B_6_ and folate (daily doses of 1 mg, 50 mg, 2.5 mg respectively) [94]. 

Frailty is characterized by muscle wasting, diminished strength, often with weight loss with or without reduced nutritional intake. Frailty is associated with an increased vulnerability to stresses, causing longer and more complicated recovery from illness or surgery [95].

Increased risk of frailty and disability has been associated with poor B vitamin status. Subjects with vitamins B_12_ and B_6_ in the lowest quintiles and subjects with elevated MMA and tHcy concentrations, have been found to have increased risk of decline in physical function and the development of frailty [96,97]. Two cross sectional studies found the length of hospital stay was associated with poor vitamin B_12_ status as assessed by MMA and serum vitamin B_12_ concentrations [98,99]. To date there are limited studies, however, if improvements in nutrition can delay frailty progression, it could significantly enhance the independence of the increasing numbers of older people.

## 13. Conclusion

Vitamin B_12_ is a particularly important vitamin for women of childbearing age and for older people, however, adequate vitamin B_12_ status over the whole of the lifecycle is needed for optimal health. There has been renewed interest in vitamin B_12_ since the reporting of associations between homocysteine and chronic disease, particularly vascular disease. The effects of sub-clinical deficiency are not fully known and many aspects of vitamin B_12_ absorption, bioavailability and metabolism are yet to be determined. The identification of sensitive biomarkers of vitamin B_12 _status will help elucidate the relationships between vitamin B_12_ and chronic disease, and help to identify those at risk of clinical and sub-clinical deficiency.
